# Using large language model to guide patients to create efficient and comprehensive clinical care message

**DOI:** 10.1093/jamia/ocae142

**Published:** 2024-06-25

**Authors:** Siru Liu, Aileen P Wright, Allison B Mccoy, Sean S Huang, Julian Z Genkins, Josh F Peterson, Yaa A Kumah-Crystal, William Martinez, Babatunde Carew, Dara Mize, Bryan Steitz, Adam Wright

**Affiliations:** Department of Biomedical Informatics, Vanderbilt University Medical Center, Nashville, TN 37203, United States; Department of Computer Science, Vanderbilt University, Nashville, TN 37212, United States; Department of Biomedical Informatics, Vanderbilt University Medical Center, Nashville, TN 37203, United States; Department of Medicine, Vanderbilt University Medical Center, Nashville, TN 37232, United States; Department of Biomedical Informatics, Vanderbilt University Medical Center, Nashville, TN 37203, United States; Department of Biomedical Informatics, Vanderbilt University Medical Center, Nashville, TN 37203, United States; Department of Medicine, Vanderbilt University Medical Center, Nashville, TN 37232, United States; Department of Biomedical Informatics, Vanderbilt University Medical Center, Nashville, TN 37203, United States; Department of Medicine, Vanderbilt University Medical Center, Nashville, TN 37232, United States; Department of Biomedical Informatics, Vanderbilt University Medical Center, Nashville, TN 37203, United States; Department of Medicine, Vanderbilt University Medical Center, Nashville, TN 37232, United States; Department of Biomedical Informatics, Vanderbilt University Medical Center, Nashville, TN 37203, United States; Department of Pediatric Endocrinology, Vanderbilt University Medical Center, Nashville, TN 37232, United States; Department of Medicine, Vanderbilt University Medical Center, Nashville, TN 37232, United States; Department of Medicine, Vanderbilt University Medical Center, Nashville, TN 37232, United States; Department of Biomedical Informatics, Vanderbilt University Medical Center, Nashville, TN 37203, United States; Department of Medicine, Vanderbilt University Medical Center, Nashville, TN 37232, United States; Department of Biomedical Informatics, Vanderbilt University Medical Center, Nashville, TN 37203, United States; Department of Biomedical Informatics, Vanderbilt University Medical Center, Nashville, TN 37203, United States; Department of Medicine, Vanderbilt University Medical Center, Nashville, TN 37232, United States

**Keywords:** clinical decision support, large language model, message content, patient-doctor communication, primary health care, patient portal

## Abstract

**Objective:**

This study aims to investigate the feasibility of using Large Language Models (LLMs) to engage with patients at the time they are drafting a question to their healthcare providers, and generate pertinent follow-up questions that the patient can answer before sending their message, with the goal of ensuring that their healthcare provider receives all the information they need to safely and accurately answer the patient’s question, eliminating back-and-forth messaging, and the associated delays and frustrations.

**Methods:**

We collected a dataset of patient messages sent between January 1, 2022 to March 7, 2023 at Vanderbilt University Medical Center. Two internal medicine physicians identified 7 common scenarios. We used 3 LLMs to generate follow-up questions: (1) Comprehensive LLM Artificial Intelligence Responder (CLAIR): a locally fine-tuned LLM, (2) GPT4 with a simple prompt, and (3) GPT4 with a complex prompt. Five physicians rated them with the actual follow-ups written by healthcare providers on clarity, completeness, conciseness, and utility.

**Results:**

For five scenarios, our CLAIR model had the best performance. The GPT4 model received higher scores for utility and completeness but lower scores for clarity and conciseness. CLAIR generated follow-up questions with similar clarity and conciseness as the actual follow-ups written by healthcare providers, with higher utility than healthcare providers and GPT4, and lower completeness than GPT4, but better than healthcare providers.

**Conclusion:**

LLMs can generate follow-up patient messages designed to clarify a medical question that compares favorably to those generated by healthcare providers.

## Introduction

High-quality patient-centered healthcare relies heavily on effective communication between patients and healthcare providers. In recent years, the number of patients using patient portals to communicate with their physicians has increased significantly.^1^ Patients greatly appreciate the opportunity to message with their healthcare providers^2^; however, healthcare providers often spend large amounts of administrative time responding to patient messages.^3^ Moreover, a large volume of patient messages and the extensive time required to manage these messages have been reported to be associated with provider burnout.^4^^,^^5^ The efficiency of this method of communication is in urgent need of improvement.^6^ One notable issue is that initial patient messages may sometimes lack accurate and complete contextual details, resulting in multiple rounds of messaging for physicians to gather the necessary information. For example, when a patient requests an antiviral such as nirmatrelvir-rotonavir to treat COVID-19, they might omit critical details such as the date of symptom onset, making it difficult for providers to assess their eligibility for the prescription.

Frequent back-and-forth messaging in patient portals between patients and healthcare providers is a common issue. A retrospective review of 5 million patient messages revealed that ∼30% of message threads consisted of three or more messages, and this percentage has been increasing each year.^7^ Back-and-forth messaging not only adds an additional workload to healthcare providers, but also hinders patients from taking the next step in treatment promptly. Moreover, the asynchronous nature of electronic communication may cause delays that can span weeks before full resolution. Notably, patients sometimes simply do not respond to follow-up questions from their healthcare providers, which can result in untreated conditions and barriers to patient care.

The potential of large language models (LLMs) to improve the efficiency of patient-provider messaging has been initially investigated, especially in drafting replies from clinicians to patients. A study that compared responses generated by ChatGPT with those from physicians to 195 patient questions found that ChatGPT responses were rated higher in terms of quality and empathy.^8^ In our previous research, we fine-tuned a LLM with patient-provider messaging data at Vanderbilt University Medical Center (VUMC) and generated replies that were rated positively for both empathy and accuracy.^9^ The utility of LLMs to generate follow-up questions that aid patients in creating efficient and comprehensive initial messages, thus minimizing the need for back-and-forth information exchange, is not yet understood.

This study aims to investigate the feasibility of using LLMs to engage with patients at the time they are drafting a question to their healthcare providers, and generate pertinent follow-up questions that the patient can answer before sending their message, with the goal of ensuring that their healthcare provider receives all the information they need to safely and accurately answer the patient’s question, eliminating back-and-forth messaging and the associated delays and frustrations.

## Methods

**Figure 1.**
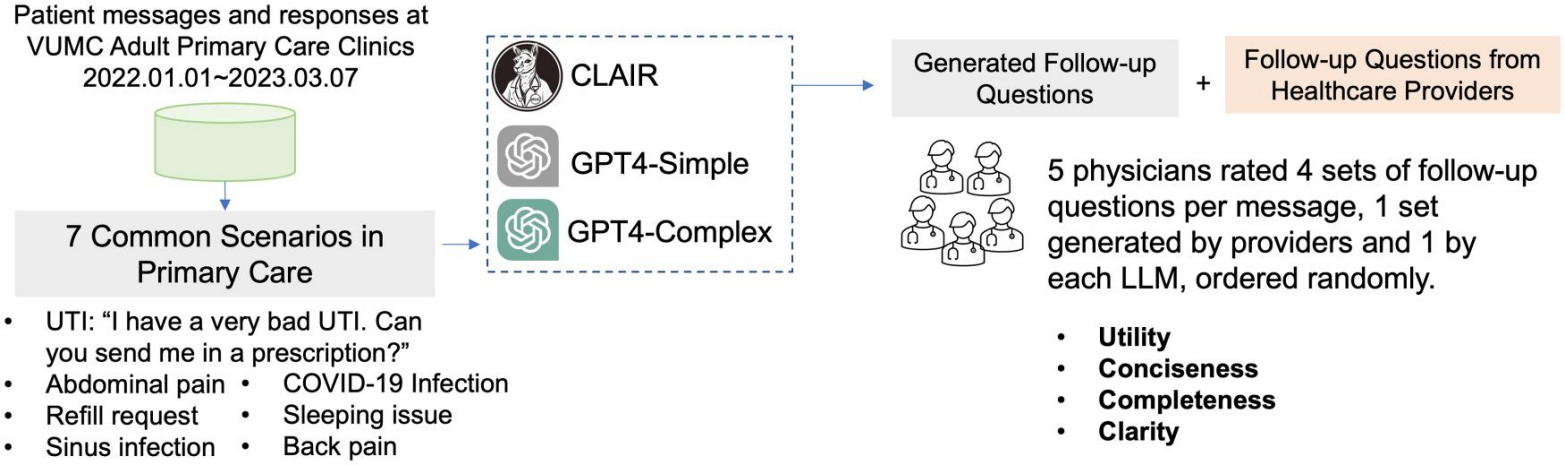
Study overview. Abbreviations: UTI = urinary tract infection; CLAIR = comprehensive LLM artificial intelligence responder.

This study was conducted at VUMC. This research was reviewed by the Vanderbilt University Institutional Review Board and found to be exempt. The study overview is displayed in Figure 1. We collected a dataset of messages sent from patients to their primary care providers (PCPs) through our patient portal, My Health at Vanderbilt^10^ along with the responses to those messages, between January 1, 2022 and March 7, 2023. To identify common patient message scenarios, we initially considered automatically counting the number of rounds of messaging. However, this approach required the development of an automatic classification model and a well-organized classification framework. Therefore, we chose to conduct interviews with internal medicine physicians (A.P.W., S.H.) to gain a better understanding of common patient message scenarios based on their past experiences in communicating with patients through My Health at Vanderbilt. Based on their input, we selected 7 representative patient message threads that involved back-and-forth communication between patients and providers. In these message threads, responses to the patient message were written by the PCP or another member of the care team (some PCPs have a provider such as a nurse who helps respond to patient messages.) We removed protected health information from the messages, but otherwise left them unchanged.

For each patient question, we generated three new sets of follow-up questions: (1) Comprehensive LLM Artificial Intelligence Responder (CLAIR): a fine-tuned LLM using patient-provider messages at VUMC, (2) GPT-simple: GPT4 with a simple prompt, and (3) GPT4-complex: GPT4 with a complex prompt, focusing on clarifying patient symptoms and checking for recent changes in the patient’s conditions. The CLAIR model was developed using the same process reported previously.^9^ We applied low-rank adaption to conduct supervised fine-tuning on Llama2 (70B) using a local dataset of patient messages and responses from their physicians at VUMC.^11^ Llama2 (70B) is a powerful LLM with 70 billion parameters and a context length of 4096.^12^ The Llama family of LLMs has been widely used as foundation models for the development of fine-tuned models, such as Med-Alpaca, which was fine-tuned using medical question-and-answer data.^13^ The prompts used in GPT4-simple and GPT4-complex were reported in Appendix S1. The GPT4 model was the OpenAI GPT-4 LLM via Microsoft Azure, which was deployed in a protected environment at VUMC to protect patient privacy.

We invited five physicians to evaluate the generated follow-up questions and follow-up questions from actual healthcare providers. Participants were blinded to whether a given response was from the human healthcare team or generated by a model. The participants reviewed the patient messages and rated the responses using a 5-point Likert scale (1-strongly disagree, 5-strongly agree) for the following metrics: (1) **Utility**: The follow-up questions would be useful to a healthcare provider in responding to the patient message, (2) **Conciseness**: All follow-up questions are necessary for a healthcare provider in addressing the patient’s concern, (3) **Completeness**: The follow-up questions are not missing important information necessary for a healthcare provider in addressing the patient’s concern [Please note that the follow-up questions are intentionally designed not to ask for information that would be better found in the electric health record (EHR), such as current medications, allergy history.], (4) **Clarity**: The follow-up questions are easy to understand and answer by patients.

For each metric, the mean and SD were reported. The overall score was determined by averaging the scores from all four metrics. For each patient message, we used the overall scores to compare the performance of generated follow-up questions. If the overall scores were the same, then the score of “utility” metric would be used to determine the final ranking. We used the Kruskal-Wallis H test^14^ with Dunn’s *post hoc* tests^15^ to compare the scores of the AI-generated follow-up questions with those from the actual healthcare providers. We set the significance level at 0.05 to determine statistical significance. In addition, we calculated the intraclass correlation coefficient (ICC) to assess rater agreement. An ICC value below 0.5 indicates low agreement, a value between 0.5 and 0.74 indicates moderate agreement, a range of 0.75-0.9 indicates high agreement, and values above 0.9 indicate very high agreement.^16^ Python 3.8 was used for statistical calculations. We also provided descriptive statistics to outline the demographics of the participants, including their specialties and years of practice.

## Results

Five physicians participated in the survey (four primary care physicians, one pediatrician). The average length of practice was 12.8 years. The ICC value was 0.76 [0.68,0.82], demonstrating high agreement.

### Top-rated follow-up questions

For each scenario, we generated potential follow-up questions using the three AI models and tasked the physicians with rating each set of follow-up questions, as well as the actual human-generated follow-up questions that had been sent to the patient by their healthcare provider. Within seven scenarios, GPT4-complex generated two sets of top-rated follow-up questions, while the remaining five sets of top-rated follow-up questions were from our CLAIR model. The top-rated follow-up questions, related patient messages and actual follow-up questions from healthcare providers are listed in Table 1. The radar chart of mean scores for the top-rated generated follow-up questions compared with the actual follow-up questions are also displayed in Table 1. All follow-up questions and patient messages are reported in Appendix S2.

**Table 1.** Top-rated follow-up questions for each selected patient message and actual questions from healthcare providers.

1. Patient: “I am having a dull pain in my abdomen under my ribs on the right side. Liver, or gallbladder, or other. I’d like to be seen. Thanks.” GPT4-Complex Please indicate if you have any symptoms: fever, jaundice (yellowing of the skin or eyes), changes in urine color, light-colored stool, nausea, vomiting, changes in appetite, weight loss, or fatigue. On a scale from 0 to 10, with 0 being no pain and 10 being the worst pain imaginable, how would you rate your abdominal pain? When did you first notice the pain under your ribs on the right side? Have you taken any over-the-counter medications to alleviate the pain? If so, which ones and have they provided any relief? Have you experienced any recent injuries or strain that might be related to your abdominal pain? Are you currently or could you possibly be pregnant? Have there been any recent changes in your bowel movements or eating habits? Have you had any similar pain in the past, and if so, was a cause determined at that time? Healthcare Provider: Do you have any other symptoms? How long has this been going on?	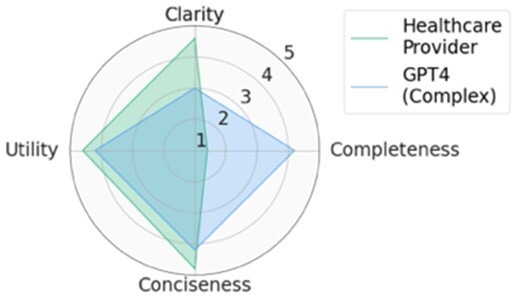

2. Patient: “Can I get a new prescription for 90 with a refill to (pharmacy name) in (town)? They say they can give me 60 but need a new prescription to give me the full 90. Thank you.” CLAIR: What medication are you requesting? Can you please confirm your current dosage? Healthcare Provider: Which medication are you referring to?	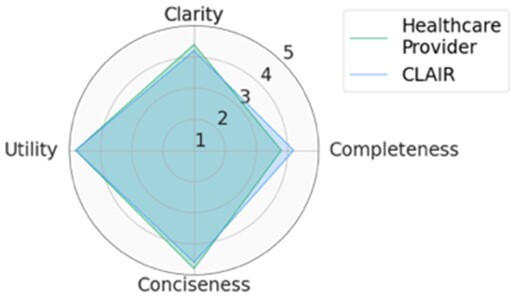

3. Patient: “Hey, I’ve got the vid I’m not too sick except my throat is killing me anything I can take? Hope you well!!!” CLAIR: Are you having any other symptoms besides the sore throat? Do you have a fever? Any shortness of breath? Are you vaccinated? If so, how many doses have you had? Are you currently in isolation? If so, for how long? What medications do you take? What does your throat feel like? Is it sore, scratchy, or something else? Healthcare Provider: Do you have strep?	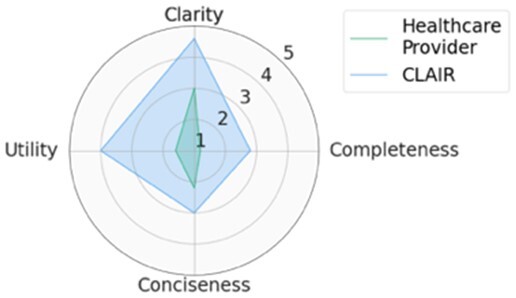

4. Patient: “Hello, ever since baby started sleeping well, I’ve suffered from insomnia every few nights. It’s starting to cause lots of fatigue and anxiety around sleep. Wondering if there’s anything to be done medically. Thanks!” CLAIR: Do you have a history of depression or anxiety? What medications have you tried for sleep? How long has this been going on? Are you having trouble falling asleep, staying asleep, or both? What is your general sleep schedule? Healthcare Provider: What have you tried?	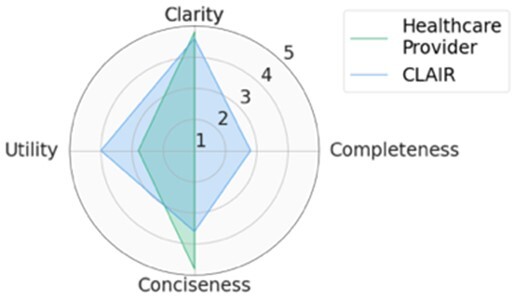

5. Patient: “I have a very bad UTI. Can you send me in a prescription?” CLAIR: What are your symptoms? Do you have a fever? How long have you been having symptoms? Do you have pain in your lower back? Are you having nausea, vomiting, or diarrhea? Have you had a UTI before? Have you had any testing done? What is your pain level? Healthcare Provider: Could you tell me about your symptoms, any fever, any back pain, pain with urination? Do you notice any blood in your urine? When did your symptoms start?	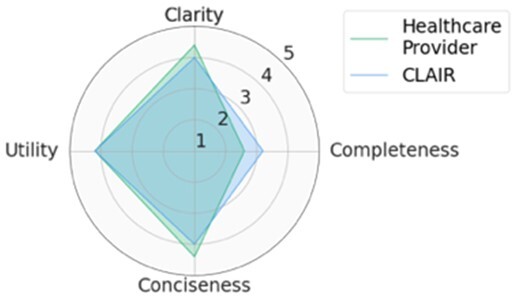


6. Patient: “Hey, Dr. X! I have a stupid sinus infection. It started out as a cold 2 1/2 weeks ago, and now its green and I have sinus pressure and pain on the right side. Can you send a Z pack to (pharmacy name) please? Thanks a bunch.” GPT4-Complex Please indicate if you have any symptoms: fever, headache, nasal congestion, decreased sense of smell or taste, cough, sore throat, fatigue, or dental pain. Has the color, consistency, or amount of nasal discharge changed recently? Have you noticed any new symptoms, such as vision changes, swelling around the eyes, or ear pain? Are you experiencing any difficulty breathing or shortness of breath? Have you taken any over-the-counter medications or remedies to alleviate your symptoms? If so, which ones and have they provided any relief? Have you had any similar infections in the past, and how were they treated? Are you currently pregnant or is there a possibility that you could be pregnant? Healthcare Provider: Are you running a fever? Are you having any shortness of breath or difficulty breathing? Have you taken any over the counter medications? Have you taken a covid test in the last 10 days?	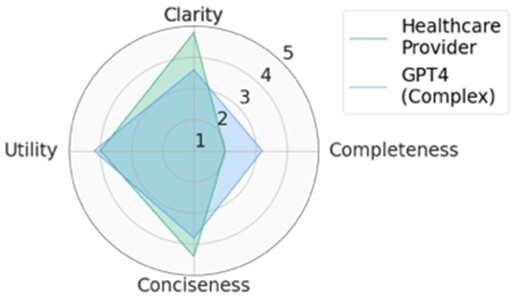

7. Patient: “I have been going through some back pain it started in my neck and right shoulder I have been nursing the pain with Advil and ibuprofen along with heat and cold pack it works for a while returns when I go to bed.” CLAIR: Can you tell me a little bit more about the pain you’re experiencing? When did it start? Where is the pain located? Does anything make it better or worse? What have you done for it so far? How painful is it on a scale of 1-10? Healthcare Provider: Would you like to start some physical therapy or a trial of a muscle relaxant? How long has this been going on? Has there been any trauma?	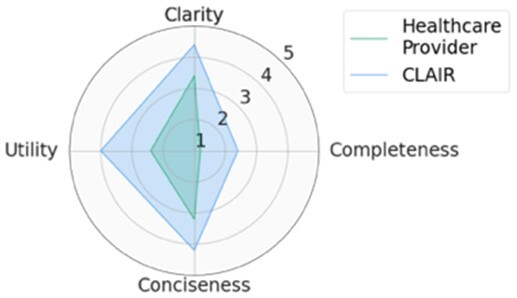

### Results of expert review of follow-up questions

**Figure 2.**
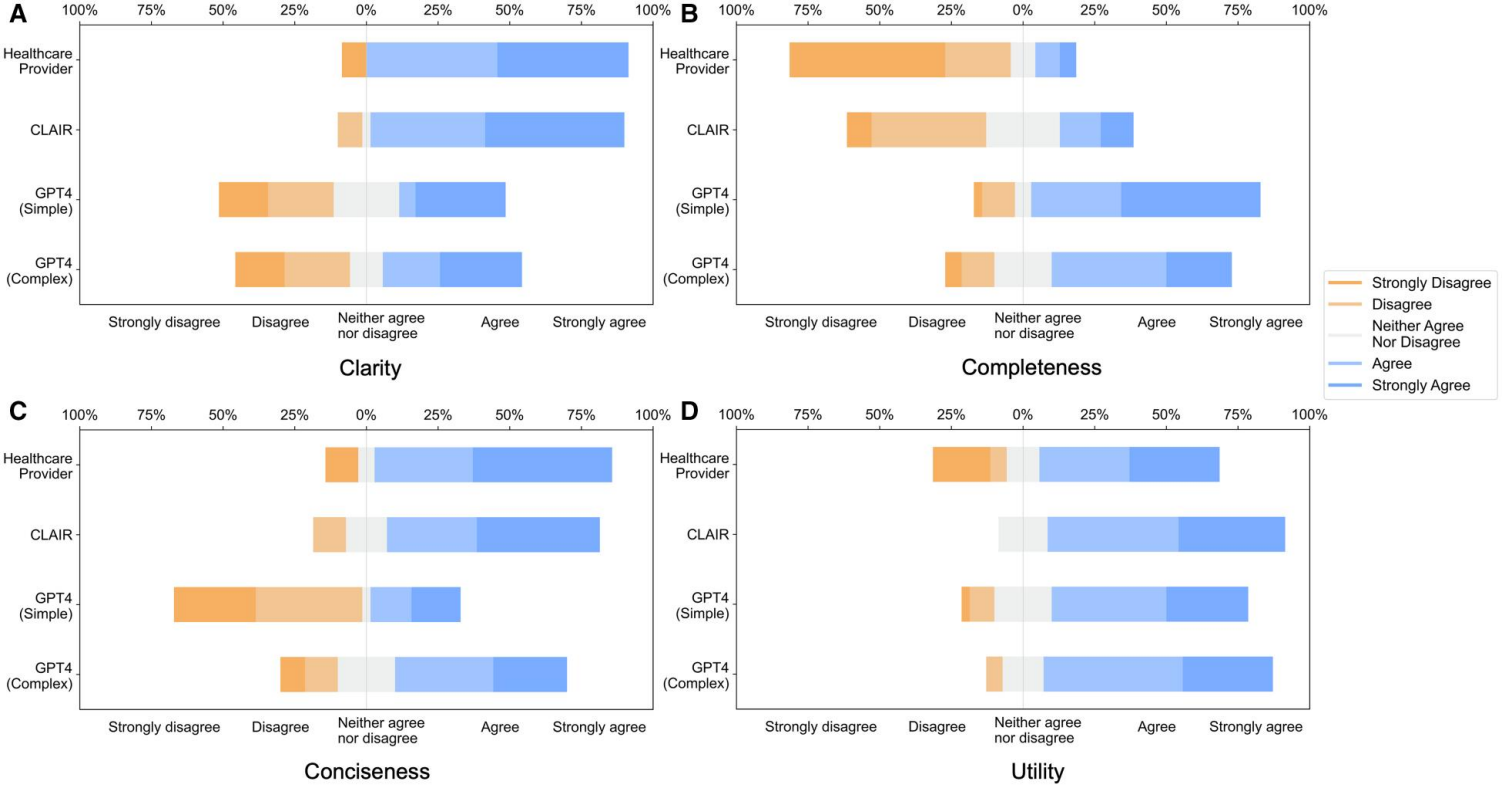
Stacked bar charts of the scores for clarity, completeness, conciseness, and utility of follow-up questions generated by healthcare providers, CLAIR, GPT4-simple, and GPT4-complex.

Provider-generated questions received high scores for clarity, conciseness, and utility but lower scores for completeness. CLAIR-generated questions received ratings comparable to provider-generated questions for clarity and conciseness, with higher utility but low completeness. Similar to CLAIR questions, those generated by GPT4-simple and GPT4-complex achieved high scores for clarity and utility. However, their ratings varied for completeness and conciseness: GPT4-simple generated questions received high scores for completeness, but lower scores for conciseness. Conversely, GPT4-complex generated questions received high scores for both completeness and conciseness. Figure 2 displays stacked bar charts illustrating the distribution of ratings for follow-up questions from healthcare providers, CLAIR, GPT4-simple, and GPT4-complex across each metric.

Significant differences (*P* < .05) were identified on clarity, completeness, and conciseness. In the *post hoc* analysis, questions from healthcare providers (4.2 ± 1.1) and CLAIR (4.3 ± 0.9) were rated significantly higher in clarity compared to those from GPT4-simple (3.1 ± 1.5) and GPT4-complex (3.2 ± 1.5). Regarding completeness, questions from healthcare providers had the lowest score (1.9 ± 1.2), significantly below the others. Among them, CLAIR-generated questions (2.8 ± 1.2) were rated significantly lower than GPT4-simple (4.1 ± 1.1) and GPT4-complex (3.6 ± 1.1). In terms of conciseness, questions from GPT4-simple received the lowest score (2.5 ± 1.5), which was significantly lower than healthcare providers (4.1 ± 1.3), CLAIR (4.1 ± 1.0), and GPT4-complex (3.6 ± 1.2). For utility, CLAIR-generated questions had the highest score (4.1 ± 0.8), while questions from healthcare providers had the lowest score (3.5 ± 1.5). Scores of the utility for GPT4-simple and GPT4-complex generated questions were 3.8 ± 1.0 and 4.1 ± 0.8, respectively. However, no significant difference in utility was identified. Means and SD of rating scores are listed in Table 2.

**Table 2.** Means and SD for survey questions rated on a 5-point Likert scale, with 1 indicating “strongly disagree” and 5 indicating “strongly agree.”

Model	Clarity	Completeness	Conciseness	Utility
**Healthcare Provider**	4.2 ± 1.1	1.9 ± 1.2	4.1 ± 1.3	3.5 ± 1.5
**CLAIR**	4.3 ± 0.9	2.8 ± 1.2^a^	4.1 ± 1.0	4.2 ± 0.7
**GPT4-Simple**	3.1 ± 1.5^a^^,^^b^	4.1 ± 1.1^a^^,^^b^	2.5 ± 1.5^a^^,^^b^	3.8 ± 1.0
**GPT4-Complex**	3.2 ± 1.5^a^^,^^b^	3.6 ± 1.1^a^^,^^b^	3.6 ± 1.2^c^	4.1 ± 0.8

Results are denoted by

a if models had a significant effect relative to Healthcare Provider,

b if significant to CLAIR,

c if significant to GPT4-Simple at the *P* = .05 using Kruskal-Wallis H test with Dunn’s *post hoc* tests.

## Discussion

In this study, we investigated the feasibility of using LLMs to generate follow-up questions for patients who are writing messages to healthcare providers with a goal of helping providers answer the patients’ questions the first time and reduce back-and-forth messages. The GPT4 model generated more useful and complete, but less clear, follow-up questions than those written by healthcare providers. The CLAIR model generated follow-up questions with similar clarity and conciseness as the questions from healthcare providers, with higher utility than healthcare providers and GPT4 models, and lower completeness than the GPT4, but better than healthcare providers.

Our approach of using LLM to generate questions for patients differs from current LLM research on patient messages that focuses on drafting replies for healthcare providers. For example, Epic now allows healthcare organizations to design prompts to generate draft replies using the GPT4 model.^17^ Our approach has several advantages. First, patients may omit important information when messaging their healthcare provider—this difficulty is not addressed by LLM draft response generators. Second, follow-up questions can be adapted to the patient’s specific situation through prompt engineering to provide more personalized care, such as education, familiarity with medical terminology and the patient’s own medical history stored in the EHR.^18^ In contrast, a significant disadvantage of using LLM to draft replies for healthcare providers is that it may disturb their established communication routines. Many healthcare providers have their own styles of replying to patient messages, and some may prefer to see patients in person or call them. In addition, the replies generated by LLMs are usually longer than their own replies,^8^ which can lead to extra time reviewing and editing these generated drafts, thereby impacting their efficiency and workflow.

In contrast to the advantages of our approach, displaying LLM generated questions to patients may increase the length of patient messages, thus increasing the workload of healthcare providers (although it may also reduce it since the information the patient provides may be useful and therefore reduce the effort of the provider). Future work could use artificial intelligence to identify patient messages that are more appropriate for an “eVisit” (an asynchronous virtual visit with a healthcare provider using an online patient portal) or telemedicine or in-person encounter rather than simply responding to the message. Furthermore, considering the robust summarization capabilities of LLMs,^19^ future research could investigate the potential of using LLM to summarize long patient messages in order to highlight important information to healthcare providers.

### Limitations

Several limitations exist in this study. First, we evaluated the follow-up questions from physicians’ perspectives. Future research could explore patients’ perspectives to evaluate the utility of the generated follow-up questions in helping them craft messages to their PCPs. This could involve gathering patient feedback on their attitudes and ratings of generated following questions based on metrics, such as clarity, completeness, and conciseness. Second, we compared follow-up questions generated from GPT4 and a locally fine-tuned LLM. Embedding external clinical knowledge (eg, clinical guidelines in UpToDate) through fine-tuning or retrieval-augmented generation might improve the performance of generated follow-up questions. Third, the clinical impact of using follow-up questions to guide patients writing their messages is unclear. As a next step, we will implement a tool to present follow-up questions in our patient portal and evaluate its impact on clinician behavior and patients.

## Conclusion

Patient messages sent to clinicians through patient portals often lack important details, resulting in multiple rounds of messages for clinicians to gather the necessary information. Our study demonstrates that LLM can be used to generate helpful follow-up questions as patients compose their messages, showing a great potential for improving patient-provider communication.

## Supplementary Material

ocae142_Supplementary_Data

## Data Availability

The patient messages and follow-up questions were reported in the Supplementary Appendix.
