# Supplementary material for: Using large language model to guide patients to create efficient and comprehensive clinical care message
Source: J Am Med Inform Assoc. 2024 Jun 25;31(8):1665–70. doi: 10.1093/jamia/ocae142 (PMC11258400; doi:10.1093/jamia/ocae142)
Supplement: ocae142_Supplementary_Data [file ocae142_supplementary_data.docx]

Appendix

Appendix 1. The simple prompt and complex prompt used in GPT4 model.

Prompt

GPT4 (Azure):

Generate a list of follow-up questions that a primary care physician might ask an adult patient, based on the patient's message. The questions should be clear, easy for patients to understand, and focused on gathering the most important and necessary information for the physician to proceed with the patient's care. Exclude introductory and concluding paragraphs, and ensure the questions are concise and directly related to the patient's message. Remove duplicate questions and only list important questions. Assume the physician has the access to the patient’s full electronic medical record, so avoid asking questions that would be better found in the medical record.

GPT4 (Azure) complex:

Generate a set of follow-up questions that a primary care physician might ask an adult patient. Assume the physician has the access to the patient’s full electronic medical record, so avoid asking questions that would be better found in the medical record. For example, do not ask about age, allergy history, side effects, medications, diagnosis, medical procedures, medical conditions, hospitalization, clinic visits etc.

Questions should be specific and easy for patients to understand. Avoid asking general questions, such as any new medical information, any recent health changes, or any other information.

If the patient message relates to medication refills, then focus only on the most important information, such as the name, strength, and dosage of the medication, and the reason for the change in prescription (if applicable). Avoid asking any less important questions. Avoid asking for the name and location of the pharmacy.

For other requests, the categories should be flexible to cover a wide range of patient concerns, such as:

1.     Specific Details: Clarify the nature of the patient’s symptoms. List all relevant symptoms, especially those associated with high-risk conditions, complications, or high-level infectious. Symptom descriptions should be clear, used with simple terms, and easy to understand for patients without medical knowledge. Symptoms should be listed in a sentence, starting with “Please indicate if you have any symptoms: …” Ensure that all indicators of common and serious conditions are included.  Avoid referring to reasoning processes or providing a diagnosis. Ask about the pain scale (if applicable) and the onset date of the symptoms.

2.    Status, Changes, and Contextual Information: Check for recent progress or changes in the patient's condition. Collect relevant information not found in the EHR but needed to address questions in the patient message. Examples include pregnancy status (for women), use of over-the-counter medications (if applicable), and specific occupational exposures linked to certain diseases (e.g., silicosis). Avoid asking questions about lifestyle, environment, work conditions, daily activities, future diseases management, previous diagnosis, or other health issues.

Ensure that each question is directly related to the patient's initial inquiry, covering all necessary aspects for a comprehensive understanding of their situation. Remove redundant questions from all categories. Exclude introductory and concluding paragraphs.

Appendix 2. Patient messages and follow-up questions.

**1. I am having a dull pain in my abdomen under my ribs on the right side. Liver, or gallbadder, or other. I’d like to be seen. Thanks**

Healthcare provider

- Do you have any other symptoms?
- How long has this been going on?

CLAIR:

- Can you describe the pain? Is it a dull constant pain? Or intermittent in nature? Is it associated with eating? Is it associated with any other symptoms?
- Do you have any other symptoms? Nausea, vomiting, fever, diarrhea, cough, shortness of breath?
- Do you have a history of gallbladder issues?

GPT4-Simple

- How long have you been experiencing this pain?
- Can you describe the pain intensity on a scale from 1 to 10?
- Does the pain radiate or spread to any other area of your body?
- Have you noticed anything that makes the pain better or worse, such as eating certain foods or physical activity?
- Are you experiencing any other symptoms, like fever, nausea, vomiting, or changes in your bowel movements?
- Have you had any similar pain in the past?
- Do you have any known allergies or intolerances to foods or medications?
- Are you currently taking any medications, supplements, or herbal remedies?
- Have you had any recent injuries or trauma to your abdomen?
- Is there a history of liver or gallbladder disease in your family?
- Have you noticed any changes in the color of your urine or stool?
- Do you consume alcohol, and if so, how frequently and how much?
- Have you experienced any recent weight loss or weight gain without trying?
- Have you been exposed to anyone with similar symptoms or diagnosed with a liver or gallbladder condition?
- When would you be available to come in for an examination?

GPT4-Complex

- Please indicate if you have any symptoms: fever, jaundice (yellowing of the skin or eyes), changes in urine color, light-colored stool, nausea, vomiting, changes in appetite, weight loss, or fatigue.
- On a scale from 0 to 10, with 0 being no pain and 10 being the worst pain imaginable, how would you rate your abdominal pain?
- When did you first notice the pain under your ribs on the right side?
- Have you taken any over-the-counter medications to alleviate the pain? If so, which ones and have they provided any relief?
- Have you experienced any recent injuries or strain that might be related to your abdominal pain?
- Are you currently or could you possibly be pregnant?
- Have there been any recent changes in your bowel movements or eating habits?
- Have you had any similar pain in the past, and if so, was a cause determined at that time?

**2. I have a very bad UTI. Can you send me in a prescription?**

Healthcare provider

- Could you tell me about your symptoms, any fever, any back pain, pain with urination?
- Do you notice any blood in your urine?
- When did your symptoms start?

CLAIR:

- What are your symptoms? Do you have a fever? How long have you been having symptoms? Do you have pain in your lower back? Are you having nausea, vomiting, or diarrhea?
- Have you had a UTI before? Have you had any testing done?
- What is your pain level?

GPT4-Simple

- When did you first notice symptoms of your UTI?
- Can you describe the symptoms you're experiencing?
- Are you experiencing any pain or burning sensation when you urinate?
- Do you feel the need to urinate more frequently than usual?
- Have you noticed any blood in your urine?
- Have you had a fever or any other systemic symptoms, like chills or nausea?
- Have you had any lower back or flank pain?
- Are you currently experiencing any vaginal discharge or irritation?
- Have you had UTIs in the past, and if so, how often?
- How have you been managing your symptoms so far?
- Are you allergic to any medications, including antibiotics?
- Are you currently taking any other medications, vitamins, or supplements?
- Do you have any other health conditions that we should be aware of?
- Are you pregnant or is there any chance you could be pregnant?
- Have you tried any over-the-counter remedies for your symptoms?
- Have you been drinking plenty of fluids?
- Have you engaged in any activities recently that might have contributed to your symptoms, such as sexual activity or using certain products in the genital area?

GPT4-Complex

- Please indicate if you have any symptoms: burning sensation during urination, frequent urge to urinate, cloudy or strong-smelling urine, pelvic pain in women, rectal pain in men, fever, or back pain.
- Have you noticed blood in your urine or any changes in urine color?
- How long have you been experiencing these symptoms?
- On a scale from 0 to 10, how would you rate your pain or discomfort?
- Have you taken any over-the-counter medications or remedies for your symptoms? If so, what have you taken and has it helped?
- Are you experiencing any nausea, vomiting, or chills alongside your symptoms?
- For women: Are you currently pregnant or is there any chance you could be pregnant?

**3. Can I get a new prescription for 90 with a refill to (pharmacy name) in (town)? They say they can give me 60 but need a new prescription to give me the full 90. Thank you.**

Healthcare Provider:

- Which medication are you referring to?

CLAIR:

- What medication are you requesting?
- Can you please confirm your current dosage?

GPT4-Simple:

- What medication are you requesting to be refilled for 90 tablets?
- How many times a day are you currently taking the medication?
- Have you experienced any new or worsening symptoms since your last prescription?
- Are you having any side effects from this medication?
- Have there been any changes in your health or other medications since your last refill?
- Is the pharmacy requesting a 90-day supply for maintenance of a chronic condition?
- Have you been taking this medication consistently and as prescribed?
- When did you last visit a healthcare provider for a check-up or review of this medication?
- Do you have enough medication to last until the new prescription is filled?
- Can you confirm the name and location of the pharmacy where you would like the prescription sent?

GPT4-Complex:

- Please provide the name, strength, and dosage of the medication you are requesting for the 90-day supply.
- Has there been any change in your condition or any new symptoms since your last prescription that would warrant a change in medication or dosage?

**4. Hey, Dr. X! I have a stupid sinus infection. It started out as a cold 2 1/2 weeks ago, and now its green and I have sinus pressure and pain on the right side. Can you send a Z pack to (pharmacy name) please? Thanks a bunch.**

Healthcare Provider

- Are you running a fever?
- Are you having any shortness of breath or difficulty breathing?
- Have you taken any over the counter medications?
- Have you taken a covid test in the last 10 days?

CLAIR:

- What other symptoms are you having? Are you having a fever? Are you having headaches?
- What other medications are you taking?

GPT4-Simple

- How severe is your sinus pain on a scale from 1 to 10, with 10 being the most painful?
- Are you experiencing any fever or chills?
- Have you noticed any changes in your vision or swelling around your eyes?
- Do you have any allergies to medications, especially antibiotics?
- Have you taken any over-the-counter medications or remedies for your symptoms, and if so, what and how long have you been taking them?
- Are you having any difficulty breathing through your nose, or have you noticed a reduced sense of smell or taste?
- Have you had any previous episodes of sinus infections, and were they treated with antibiotics?
- Are you experiencing any tooth pain or discomfort that could be related to your sinus issues?
- Have you had any unusual nasal discharge besides the green mucus, such as blood-tinged discharge?
- Are you currently experiencing any other symptoms that haven't been mentioned, such as ear pain or a sore throat?
- Are there any health conditions or recent changes in your health that we should be aware of since your last visit?

GPT4–Complex

- Please indicate if you have any symptoms: fever, headache, nasal congestion, decreased sense of smell or taste, cough, sore throat, fatigue, or dental pain.
- Has the color, consistency, or amount of nasal discharge changed recently?
- Have you noticed any new symptoms, such as vision changes, swelling around the eyes, or ear pain?
- Are you experiencing any difficulty breathing or shortness of breath?
- Have you taken any over-the-counter medications or remedies to alleviate your symptoms? If so, which ones and have they provided any relief?
- Have you had any similar infections in the past, and how were they treated?
- Are you currently pregnant or is there a possibility that you could be pregnant?

**5. Hey, I’ve got the vid I’m not too sick except my throat is killing me anything I can take? Hope you well!!!**

Healthcare Provider

Are we sure you also don’t have strep?

CLAIR:

- Are you having any other symptoms besides the sore throat? Do you have a fever? Any shortness of breath?
- Are you vaccinated? If so, how many doses have you had?
- Are you currently in isolation? If so, for how long?
- What medications do you take?
- What does your throat feel like? Is it sore, scratchy, or something else?

GPT4-Simple

- When did you first start feeling ill?
- Can you describe the severity of your throat pain on a scale of 1 to 10?
- Are you experiencing any difficulty swallowing or breathing?
- Do you have any other symptoms, such as fever, cough, or body aches?
- Have you taken your temperature, and if so, what was it?
- Have you had a COVID-19 test, and was it positive?
- Are you currently on any medications, including over-the-counter remedies?
- Do you have any known allergies to medications?
- Have you been in contact with anyone who has COVID-19 recently?
- Are you currently self-isolating, and for how long have you been doing so?
- Do you have any chronic health conditions or a compromised immune system?
- Are you staying hydrated and are you able to eat and drink normally?
- Have you tried any home remedies or treatments for your sore throat yet?
- Are you up-to-date with COVID-19 vaccinations and boosters?
- Do you have access to a pharmacy where you can pick up a prescription if necessary?

GPT4-Complex

- Please indicate if you have any symptoms: fever, cough, shortness of breath, loss of taste or smell, fatigue, muscle or body aches, headache, congestion or runny nose, nausea or vomiting, diarrhea.
- On a scale from 0 to 10, how would you rate your throat pain?
- When did you first notice your sore throat starting to bother you?
- Have you taken any over-the-counter medications or home remedies for your sore throat? If so, what have you taken and has it provided any relief?
- Are you experiencing any difficulty swallowing or breathing due to your sore throat?
- Have you been in close contact with anyone who has tested positive for COVID-19 in the past 14 days?
- Are you currently pregnant or is there a chance you could be pregnant?
- Have you noticed any white patches on your tonsils or any other changes inside your mouth?
- Have you had a fever in the last 24 hours, and if so, how high did it go?
- Are you able to stay hydrated and are you drinking plenty of fluids?

**6. Hello, ever since baby started sleeping well, I’ve suffered from insomnia every few nights. It’s starting to cause lots of fatigue and anxiety around sleep. Wondering if there’s anything to be done medically. Thanks!**

Healthcare Provider

What have you tried?

CLAIR:

- Do you have a history of depression or anxiety?
- What medications have you tried for sleep?
- How long has this been going on?
- Are you having trouble falling asleep, staying asleep, or both?
- What is your general sleep schedule?

GPT4-Simple

- Can you describe what you mean by insomnia? Are you having trouble falling asleep, staying asleep, or waking up too early?
- How many nights a week do you experience insomnia, and how long has this been going on?
- Approximately how many hours of sleep are you getting each night?
- Do you notice any patterns or triggers that seem to contribute to your sleepless nights?
- Have there been any recent changes in your life or stressors that might be affecting your sleep?
- How does your insomnia affect your daily activities and responsibilities?
- Do you consume caffeine, alcohol, or nicotine, and if so, how much and how close to bedtime?
- What is your bedtime routine like? Do you engage in any activities that might interfere with your sleep, such as using electronic devices or doing work in bed?
- Have you tried any home remedies or over-the-counter medications to help with your sleep? If so, what were they and did they help?
- Are you currently taking any prescription medications or supplements, and have there been any recent changes to these?
- Have you experienced any mood changes, such as feeling depressed or anxious, aside from the anxiety around sleep?
- Do you have any other symptoms that may be associated with your sleep problems, such as snoring, gasping for air, or restless legs?
- Is there a family history of sleep disorders or other relevant medical conditions?
- What is your level of physical activity, and do you exercise regularly?
- Are you experiencing any pain or physical discomfort at night that might be disrupting your sleep?
- Would you be interested in exploring non-medical interventions, such as cognitive-behavioral therapy for insomnia (CBT-I), relaxation techniques, or lifestyle modifications?
- Have you noticed any impact on your work performance or personal relationships due to lack of sleep or fatigue?
- Is there anything specific you would like to address or any particular concerns you have about potential treatments for your insomnia?

GPT4-Complex

- Please indicate if you have any symptoms: difficulty falling asleep, waking up frequently during the night, waking up too early and not being able to fall back asleep, feeling tired upon waking, or experiencing daytime sleepiness.
- On a scale from 0 to 10, with 10 being the most severe, how would you rate your fatigue on the days following your insomnia?
- When did you first start experiencing insomnia?
- Have you noticed any specific factors or conditions that seem to trigger your insomnia, such as stress or consumption of caffeine or alcohol?
- Are you currently using any over-the-counter medications or supplements to help with your sleep?
- Have you tried any strategies so far to improve your sleep, such as establishing a regular bedtime routine or creating a more comfortable sleep environment?
- Are you experiencing any other symptoms of anxiety, such as constant worry, restlessness, or difficulty concentrating, during the days or nights you have trouble sleeping?

**7. I have been going through some back pain it started in my neck and right shoulder I have been nursing the pain with Advil and ibuprofen along with heat and cold pack it works for a while returns when I go to bed.**

Healthcare Provider:

- Would you like to start some physical therapy or a trial of a muscle relaxant?
- How long has this been going on?
- Has there been any trauma?

CLAIR:

- Can you tell me a little bit more about the pain you're experiencing? When did it start?
- Where is the pain located?
- Does anything make it better or worse? What have you done for it so far?
- How painful is it on a scale of 1 - 10?

GPT4-Simple

- Can you describe the nature of your back pain (sharp, dull, throbbing, constant, intermittent)?
- When did the pain first begin?
- Have you experienced any recent injuries or engaged in activities that could have contributed to your pain?
- On a scale from 1 to 10, how would you rate the pain at its worst?
- Does the pain radiate or spread to any other parts of your body?
- Is there anything that seems to trigger the pain or make it worse (e.g., certain movements, sitting for long periods)?
- Have you noticed any numbness, tingling, or weakness in your arms or legs?
- Are there any activities or positions that relieve your pain?
- How is the pain affecting your daily activities and sleep?
- Have you taken any over-the-counter medications besides Advil and ibuprofen, or been prescribed any other treatment for your pain?
- Are you experiencing any other symptoms, such as fever, weight loss, or changes in bowel/bladder function?
- Do you have any history of chronic illnesses or previous back problems?
- Have you noticed any changes in your ability to perform routine tasks?
- Is there a family history of spinal or musculoskeletal issues?
- Are you currently under any stress or have you experienced any recent life changes that may be contributing to your pain?

GPT4-Complex

- Please indicate if you have any symptoms: numbness or tingling in your arms or fingers, weakness in your arms or hands, loss of coordination, problems with walking or balance, or severe headaches.
- On a scale from 0 to 10, with 0 being no pain and 10 being the worst pain imaginable, how would you rate your back, neck, and shoulder pain?
- Can you specify the date when your neck and shoulder pain began?
- Has the pain been constant, or does it come and go? How often does the pain return when it subsides?
- Have you experienced any recent injuries or engaged in activities that might have contributed to your back pain?
- Are you experiencing any difficulty with your daily activities due to the pain?
- Have you noticed any improvement or worsening of the pain since it started, despite the use of Advil, ibuprofen, and heat/cold packs?
- Are you currently taking any over-the-counter medications other than Advil and ibuprofen? If so, please list them.
- For women: Is there any possibility that you could be pregnant?

Appendix 3. The post-hoc analysis: Dunn's test.

Conciseness

|  | **Healthcare Provider** | **CLAIR** | **GPT4-Simple** | **GPT4-Complex** |
| --- | --- | --- | --- | --- |
| **Healthcare Provider** | 1.00 | 0.78 | **<0.001** | 0.07 |
| **CLAIR** | 0.78 | 1.00 | **<0.001** | 0.12 |
| **GPT4-Simple** | <0.001 | <0.001 | 1.00 | **0.01** |
| **GPT4-Complex** | 0.07 | 0.12 | 0.01 | 1.00 |

Completeness

|  | **Healthcare Provider** | **CLAIR** | **GPT4-Simple** | **GPT4-Complex** |
| --- | --- | --- | --- | --- |
| **Healthcare Provider** | 1.00 | **0.01** | **<0.001** | **<0.001** |
| **CLAIR** | 0.01 | 1.00 | **<0.001** | **0.02** |
| **GPT4-Simple** | <0.001 | <0.001 | 1.00 | 0.16 |
| **GPT4-Complex** | <0.001 | 0.02 | 0.16 | 1.00 |

Clarity

|  | **Healthcare Provider** | **CLAIR** | **GPT4-Simple** | **GPT4-Complex** |
| --- | --- | --- | --- | --- |
| **Healthcare Provider** | 1.00 | 0.83 | **<0.001** | **0.01** |
| **CLAIR** | 0.83 | 1.00 | **<0.001** | **<0.001** |
| **GPT4-Simple** | <0.001 | <0.001 | 1.00 | 0.86 |
| **GPT4-Complex** | 0.01 | <0.001 | 0.86 | 1.00 |
